# Semicircular canal size constrains vestibular function in miniaturized frogs

**DOI:** 10.1126/sciadv.abn1104

**Published:** 2022-06-15

**Authors:** Richard L. Essner, Rudá E. E. Pereira, David C. Blackburn, Amber L. Singh, Edward L. Stanley, Mauricio O. Moura, André E. Confetti, Marcio R. Pie

**Affiliations:** 1 Department of Biological Sciences, Southern Illinois University Edwardsville , Edwardsville, IL, USA.; 2 Programa de Pós-Graduação em Zoologia, Universidade Federal do Paraná, Curitiba, Paraná, Brazil.; 3 Florida Museum of Natural History, University of Florida, , Gainesville, FL, USA.; 4 Departamento de Zoologia, Universidade Federal do Paraná, Curitiba, Paraná, Brazil.; 5 Mater Natura—Instituto de Estudos Ambientais, Curitiba, Paraná, Brazil.; 6 Department of Biology, Edge Hill University, Ormskirk, Lancashire, UK.

## Abstract

Miniaturization has evolved repeatedly in frogs in the moist leaf litter environments of rainforests worldwide. Miniaturized frogs are among the world’s smallest vertebrates and exhibit an array of enigmatic features. One area where miniaturization has predictable consequences is the vestibular system, which acts as a gyroscope, providing sensory information about movement and orientation. We investigated the vestibular system of pumpkin toadlets, *Brachycephalus* (Anura: Brachycephalidae), a clade of miniaturized frogs from Brazil. The semicircular canals of miniaturized frogs are the smallest recorded for adult vertebrates, resulting in low sensitivity to angular acceleration due to insufficient displacement of endolymph. This translates into a lack of postural control during jumping in *Brachycephalus* and represents a physical constraint resulting from Poiseuille’s law, which governs movement of fluids within tubes.

## Teaser

Pumpkin toadlets lack postural control during jumping due to a physical constraint imposed by semicircular canal size.

## INTRODUCTION

**Fig. 1.**
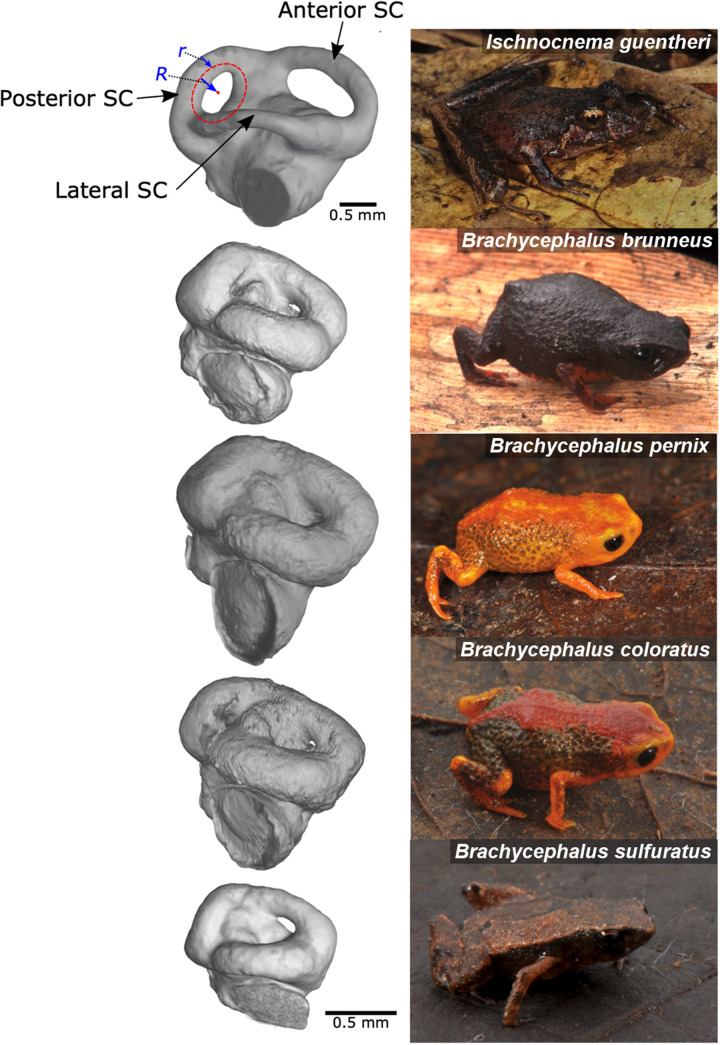
Microcomputed tomography (microCT) imaging of the inner ear of *Ischnocnema guentheri*, *B. brunneus*, *B. pernix*, *B. coloratus*, and *B. sulfuratus*. (Photographs by L.F. Ribeiro). The semicircular canals (SC) are oriented orthogonally to detect angular acceleration along three axes of motion (two vertical and one horizontal). Endolymph moves through the membranous duct within the semicircular canals. Lumen radius (*r*) was estimated as the distance from the outer margin of the endocast to the center of the endocast at its narrowest point (minimum circumference). This measurement represents the maximum possible value of *r* based on dimensions of the bony labyrinth, rather than the membranous duct which was unavailable. Circuit radius (*R*) was estimated by fitting a curve to a series of points placed along the midpoint of the entire canal (red dashed line) and measuring the distance to the center of the torus.

The vertebrate vestibular system is a network of fluid-filled chambers and canals within the bony labyrinth of the inner ear that is responsible for the sense of balance and spatial orientation. It includes otolith organs, which detect gravitational and linear acceleration, and three orthogonal semicircular canals, which detect angular acceleration (Fig. 1). Movement of the head causes endolymph, a fluid similar in density to water, to move past sensory hair cells in the ampulla, mechanically deflecting them and initiating a nerve impulse (*1*). This sensory information is directed to the central nervous system where it is used to control posture and movement through motor output to skeletal muscle.

As endolymph flows through the tubular semicircular canals, its movement is governed by Poiseuille’s law, which states that the flow (*Q*) of a fluid through a tube is directly proportional to the fourth power of the tube radius (*r*^4^), and the pressure difference between the inflow (*P*_i_) and outflow (*P*_o_) of the tube and inversely proportional to the tube length (*l*) and the fluid viscosity (η): $Q=\frac{\pi (Pi-Po)(r^{4})}{8\eta l}$ (*2*). Resistance to flow results from friction of the fluid moving past the walls of the tube, as well as fluid viscosity and turbulence. Increasing tube length or fluid viscosity decreases flow and increases resistance, whereas increasing tube radius translates into proportionally less fluid encountering the tube walls, reducing resistance.

This phenomenon is a well-known feature of the vertebrate circulatory system in which large blood vessels such as the aorta experience high flow rates due to low resistance, compared to small capillaries, which have low flow rates due to high resistance. This enables oxygenated blood to be delivered quickly to where it is needed, while allowing sufficient time for gas exchange to occur in the capillaries where resistance causes blood flow to slow markedly. Poiseuille’s law presents a potential problem for the flow of fluid within the smallest vertebrates. Their small bodies place a limit on the size—and possibly the function—of their anatomical systems (*3*), such as the size of the semicircular canals and their sensitivity to changes in angular acceleration during locomotion (*2*, *4*, *5*).

Vertebrate semicircular canals are under a remarkable size constraint. Even species varying in body mass over orders of magnitude have similarly sized semicircular canals (*6*, *7*). For example, baleen whales have semicircular canals that are only of average size for mammals (*2*). Across vertebrates, semicircular canal size is weakly dependent on body mass, except in the smallest forms in which it is limited by head size (*2*).

Two quantities that determine the geometry of the circuits formed by the semicircular canals and therefore their role in shaping the dynamics of endolymph flow are the circuit radius (*R*) and the lumen radius (*r*) (*2*). Circuit radius is the radius of the entire toroidal semicircular canal and corresponds to tube length (*l*). Lumen radius refers to the radius of the membranous duct at the center of the semicircular canal (Fig. 1). On the basis of the Poiseuille equation, reducing circuit radius should lower resistance to endolymph flow. However, this is countered by a reduction in sensitivity, measured in terms of endolymph displacement for a given stimulus (*8*, *9*). Sensitivity also declines with decreasing lumen radius, which is the dominant factor because it is raised to the fourth power in the Poiseuille equation (*2*, *5*).

**Fig. 2.**
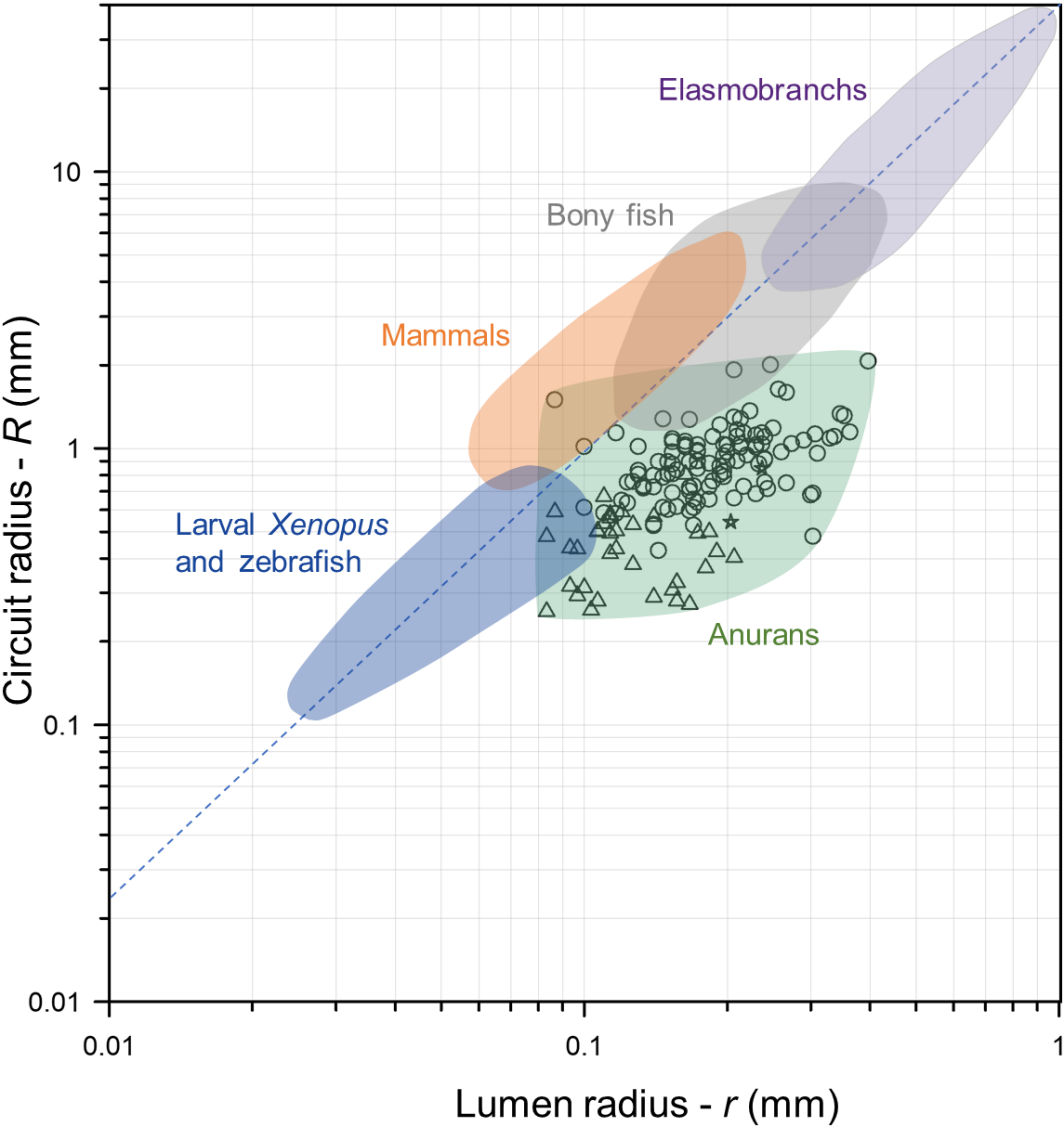
Log-log plot of lumen radius (*r*) versus circuit radius (*R*) for groups of adult and larval vertebrates [modified from (*5*)] with the canal dimensions of 147 anuran species superimposed. Values for anurans, elasmobranchs, bony fish, and mammals represent means of the anterior, posterior, and lateral semicircular canals (*2*). Measurements for larval *Xenopus* and zebrafish are based on lateral canals only. Measurements for nonanuran miniaturized taxa were unavailable and are not included. Miniaturized and small-bodied frogs <24 mm (*39*) are represented by triangles. *Ascaphus* and *Leiopelma* are represented by stars. All other frogs are represented by circles. The dashed blue line is the regression line, *R =* 38.9 × *r*^1.60^ estimated from a large sample of vertebrates (*n =* 285) (*2*).

Across vertebrates, the relationship between circuit radius and lumen radius is linear (*R* = 38.9 × *r*^1.60^; Fig. 2) (*2*). Species with a combination of small circuit and lumen radii are the least sensitive to angular acceleration. This extends to ontogenetic stages within a species, where a small head size limits the size of the semicircular canals early in development. Studies examining the relationship between semicircular canal dimensions and sensitivity to angular acceleration in larval *Xenopus* and zebrafish have used the onset of the angular vestibulo-ocular reflex (aVOR) as an important indicator of vestibular function (*4*, *5*). The aVOR reflex produces involuntary eye movement in the opposite direction of head movement and is used to stabilize gaze (*5*). The lateral semicircular canal dimensions of early ontogenetic stages of *Xenopus* (≤Nieuwkoop-Faber stage 47) place them below the generalized vertebrate aVOR reflex sensitivity threshold for detecting peak angular accelerations of 400° s^−2^ (*5*). In contrast, adult bullfrogs (*Rana catesbeiana*) are able to detect angular accelerations as low as 0.3° s^−2^, which is similar to the responses of cats and humans (*10*).

### Microtomography

The dimensions of the semicircular canals have not been investigated across a diverse array of anuran taxa varying in body size. However, there is a reason to suspect that the smallest frogs may experience size-related constraints on vestibular function, because the adult head widths (HWs) of some miniaturized species are smaller than that of *Xenopus* larvae, which are below the threshold for lateral aVOR onset (Table 1) (*4*). To assess this, we used high-resolution x-ray computed microtomographic scans (CT scans) to create three-dimensional endocasts of the inner ears of 147 anuran species from 54 families (data S1). This sample represents the full range of body size variation observed in extant anurans, from the largest frog (Goliath frog: *Conraua goliath*) to the smallest, with miniaturized representatives from 10 genera, including *Brachycephalus*.

**Table 1.** HWs for Nieuwkoop-Faber stage 47 larval *Xenopus* and the 10 smallest miniaturized anuran species included in the study.

**Species**	**ID**	**SUL (mm)**	**HW (mm)**
*Xenopus laevis* stage 47 larva	Figshare (*55*)	4.63	2.73
*Paedophryne* sp.	BPBM40153	8.06	2.32
*B. sulfuratus*	DZUP151	8.43	2.44
*Brachycephalus* *curupira*	MHNCI10285	8.63	2.63
*Paedophryne* *titan*	UMMZ242416	8.87	2.47
*B. brunneus*	MHNCI10732	9.66	2.66
*Paedophryne* *swiftorum*	BPBM31884	10.07	3.11
*B. coloratus*	MHNCI10274	10.31	2.74
*Brachycephalus* *mariatereze*	MHNCI10195	10.92	3.33
*Brachycephalus* *albolineatus*	MHNCI10295	10.95	3.14
*Sooglossus* *gardineri*	CAS156991	11.39	3.65

The dimensions of the vertical semicircular canals (i.e., anterior and posterior canals) are important variables for understanding their role in detecting angular acceleration associated with the pitch axis, a rotation that is intrinsic to anuran jumping. During takeoff, a nose-up pitching rotation elevates the anterior trunk about the hinge-like iliosacral joint to modulate jump trajectory (*11*). In the majority of frogs, this is followed by an aerial nose-down pitching rotation to position the body for landing. In contrast, rotations about the roll and yaw axes during the launch are indicative of asymmetry, as occurs with slipping or turning.

## RESULTS AND DISCUSSION

### Miniaturized frogs have the smallest semicircular canals among adult vertebrates

Our results show that the semicircular canal dimensions of miniaturized frogs (e.g., *Brachycephalus* and *Paedophryne*) are the smallest ever recorded for adult vertebrates (Fig. 2 and data S1). These and the other small frogs at the lower end of the range of semicircular canal size are thus expected to exhibit the lowest sensitivities to angular acceleration of any nonlarval vertebrate.

### Miniaturized frogs benefit from negative allometry

**Fig. 3.**
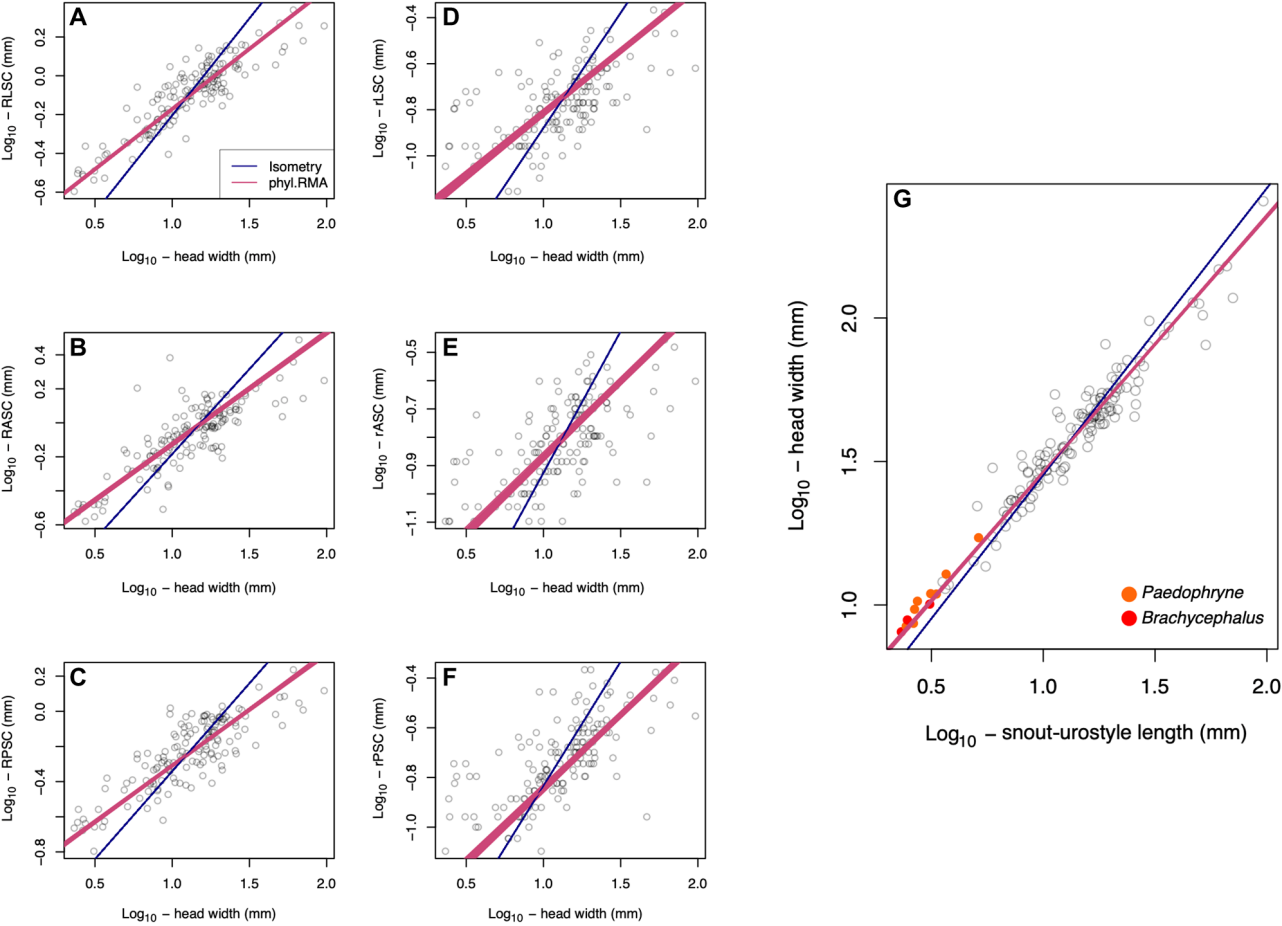
Phylogenetic RMA regression of inner ear variables versus HW and SUL versus HW for 147 anuran species. (**A** to **G**) Red line is the RMA regression line based on 100 alternative topologies. Blue line indicates simple isometry. (A) RLSC, circuit radius of lateral semicircular canal; (B) RASC, circuit radius of anterior semicircular canal; (C) RPSC, circuit radius of posterior semicircular canal; (D) rLSC, lumen radius of lateral semicircular canal; (E) rASC, lumen radius of anterior semicircular canal; (F) rPSC, lumen radius of posterior semicircular canal; (G) the two smallest miniaturized genera are represented by orange (*Paedophryne*) and red (*Brachycephalus*) circles. See Table 2 for scaling parameters.

Given the size-related physical constraints on vestibular function, we examined scaling relationships associated with the head and semicircular canals. A general negative allometry between inner ear measurements and size has been noted across a diverse array of vertebrates (*6*, *12*–*15*), and we predicted that the same trend would characterize frogs. We used phylogenetic reduced major axis (RMA) regression to assess the relationships between (i) circuit and lumen radii of the anterior, posterior, and lateral semicircular canals and HW; and (ii) HW and snout-urostyle length (SUL). We found significant negative allometry for all inner ear variables versus HW (slope < 1, *P* < 0.0001; Fig. 3, A and F). The relationship between HW and SUL was also negatively allometric (slope = 0.89, *P* < 0.0001; Table 2 and Fig. 3G). Thus, small frogs have disproportionately large semicircular canals relative to head size and large heads relative to body size. This is likely beneficial to miniaturized frogs because sensitivities are greater than what would occur under isometry (Fig. 3, A to G).

**Table 2.** Phylogenetic scaling results of the phylogenetic RMA analysis for linear measurements and SUL against HW. All measurements were log_10_ transformed before the analysis. Estimates are provided as means ± 95% quantiles across 100 alternative topologies. The *P* value indicates the statistical significance of the difference between the obtained slope with that expected under simple isometry.

**Measurements**	**Slope**	***R* ^2^**	**λ**	***P***
RASC	0.66 (0.65–0.67)	0.62 (0.60–0.64)	0.86 (0.79–0.94)	≪0.0001
RLSC	0.62 (0.62–0.63)	0.76 (0.75–0.77)	0.76 (0.70–0.84)	≪0.0001
RPSC	0.64 (0.63–0.65)	0.57 (0.56–0.59)	0.77 (0.70–0.85)	≪0.0001
rASC	0.54 (0.53–0.55)	0.41 (0.40–0.42)	0.85 (0.79–0.91)	≪0.0001
rLSC	0.54 (0.53–0.56)	0.41 (0.39–0.43)	0.87 (0.82–0.93)	≪0.0001
rPSC	0.59 (0.58–0.60)	0.49 (0.47–0.50)	0.83 (0.78–0.88)	≪0.0001
SUL	0.89 (0.88–0.90)	0.94 (0.93–0.94)	0.87 (0.83–0.92)	≪0.0001

### Pumpkin toadlets exhibit uncontrolled landings

We hypothesized that low sensitivity to angular acceleration would manifest itself in poor postural control due to the predominant role of vestibular feedback in anuran jumping. A study of jumping in experimentally manipulated cane toads (*Rhinella marina*) indicated that ablation of the vestibular system produced the greatest deficits in takeoff and landing behavior compared with loss of either visual or proprioceptive feedback. Toads with compromised vestibular systems were characterized by highly variable and uncontrolled takeoffs and landings (*16*).

We examined jumping behavior in four species of *Brachycephalus*: *Brachycephalus brunneus* (*n =* 1, 12 trials), *Brachycephalus coloratus* (*n* = 5, 58 trials), *Brachycephalus pernix* (*n* = 4, 63 trials), and *Brachycephalus sulfuratus* (*n* = 1, 5 trials), as well as a nonminiaturized species in the sister genus, *Ischnocnema henselii* (*n* = 2, 19 trials). We filmed jumping in an arena using high-speed video (movies S1 to S5). In most anurans, jumping occurs by rapidly extending hindlimbs and elevating the trunk about the iliosacral joint until they lose contact with the substrate. Hindlimbs are flexed during flight (mid-flight hindlimb recovery) in preparation for a forelimbs-first landing, after which the flexed hindlimbs are brought beneath the body in preparation for the next jump (*17*–*19*). This description was consistent with our observations of jumping in *I. henselii* (movie S1). However, it did not match our observations of the four *Brachycephalus* spp., which exhibited highly variable and uncontrolled jumps. These were typified by mid-air pitching, rolling, and yawing, often occurring simultaneously, with crash landings characterized by extended hindlimbs throughout the flight phase and delayed hindlimb recovery (movies S2 to S5).

Aerial rotation was further examined in a subset of trials in which individuals traveled at least two body lengths and showed no evidence of slipping. Excessive nose-up pitching (>45°) that continued throughout the flight phase was the most frequently observed rotation during jumping in *Brachycephalus* and was seen in over half of recorded trials (61 of 115 = 53%). Rolling (43 of 115 = 37%) and yawing (40 of 115 = 35%) rotations were also common. In many cases, these rotations resulted in the frog landing on its back (40 of 115 = 35%; movie S6).

### Angular accelerations are lowest during the flight phase

We next analyzed the kinematics of pitching rotations to determine how angular acceleration, a key component of vestibular feedback, changed throughout the jump. Most recorded trials in the four species of *Brachycephalus* were characterized by complex multiaxial rotations that were not conducive to analysis with our setup (*n* = 74). We examined a subset of trials from one species, *B. pernix* that exhibited minimal rotation about the roll and yaw axes and which did not exhibit pitching that exceeded 90° (*n* = 3 individuals, eight trials). We compared angular acceleration during the launch and flight phases and determined that mean angular acceleration was significantly higher during the launch phase (mean = 0.8 × 10^4^ ± 738° s^−2^ SEM) than during the flight phase [mean = −0.1 × 10^4^ ± 410° s^−2^ SEM; Wilcoxon signed-rank test *W*(7) = 0, two-tailed *P* < 0.001; fig. S1 and data S2], indicating that the flight phase was more likely to present detection problems for animals with low sensitivity to angular acceleration.

### Pumpkin toadlet jumping is similar to frogs and toads with vestibular ablation

We propose that the low angular accelerations observed during the flight phase in pumpkin toadlets make them unable to use vestibular feedback to control posture in preparation for landing. Further evidence for impeded vestibular feedback during jumping in *Brachycephalus* comes from studies of frogs and toads following bilateral labyrinthectomy or differential ablation of the semicircular canals (*16*, *20*, *21*). Descriptions of their jumping behavior are remarkably similar to *Brachycephalus*, including uncontrolled landings with extended hindlimbs and delayed hindlimb recovery. In addition, forelimbs continue to move normally in labyrinthectomized or ablated frogs and toads but are poorly positioned for landing (*16*, *20*, *21*). This too is consistent with our observations of *Brachycephalus* (movies S2 to S6). Forelimb movement in the absence of vestibular feedback indicates control via a nonvestibular mechanism (*16*, *21*, *22*), possibly as a reflexive response driven by proprioception resulting from hindlimb extension at the launch (*22*).

Toads (*R. marina*) with impaired vestibular systems frequently exhibited a lack of bilateral coordination and asymmetric limb movement that resulted in mid-air rolling. Many contacted the ground with their head or trunk, rather than their forelimbs, while others landed hindlimbs first (*16*). Frogs with bilateral ablation of either the anterior or posterior canals exhibited pitching rotations that in extreme cases resulted in backward somersaulting. In the absence of vestibular feedback, bilaterally ablated frogs were unable to quickly transition from the hindlimb extension phase of the launch to the hindlimb recovery phase of landing (*21*). Thus, the extended posture of *Brachycephalus* would appear to represent the default in the absence of vestibular feedback.

Uncontrolled landings and delayed hindlimb recovery also characterize jumping in the earliest diverging group of living frogs (Ascaphidae + Leiopelmatidae), which diverged from other frogs >200 million years ago and likely before the origin of controlled landings (*17*–*19*). Given the phylogenetic position of Brachycephalidae within the Neobatrachia and the normal jumping behavior of the sister genus, *Ischnocnema*, the loss of vestibular feedback is a better explanation for the jumping behavior of *Brachycephalus*, rather than retention of an ancestral trait. Our analysis of vestibular shape from CT scans confirms that the semicircular canal dimensions of Ascaphidae and Leiopelmatidae are typical for frogs of their size, allowing us to conclude that the similar performance is not due to these taxa having canals similar in size to *Brachycephalus* (Fig. 2).

### Body size predicts timing of hindlimb recovery

Last, to determine whether the jumping behavior of *Brachycephalus* provides evidence of a size-related constraint, we compared the timing of hindlimb recovery during jumping among a diverse array of frog taxa varying in body size, phylogenetic relatedness, habitat type, and locomotor performance. A total of 38 species from 23 genera representing nine families were collected from the wild and filmed with high-speed video (data S3 and S4).

**Fig. 4.**
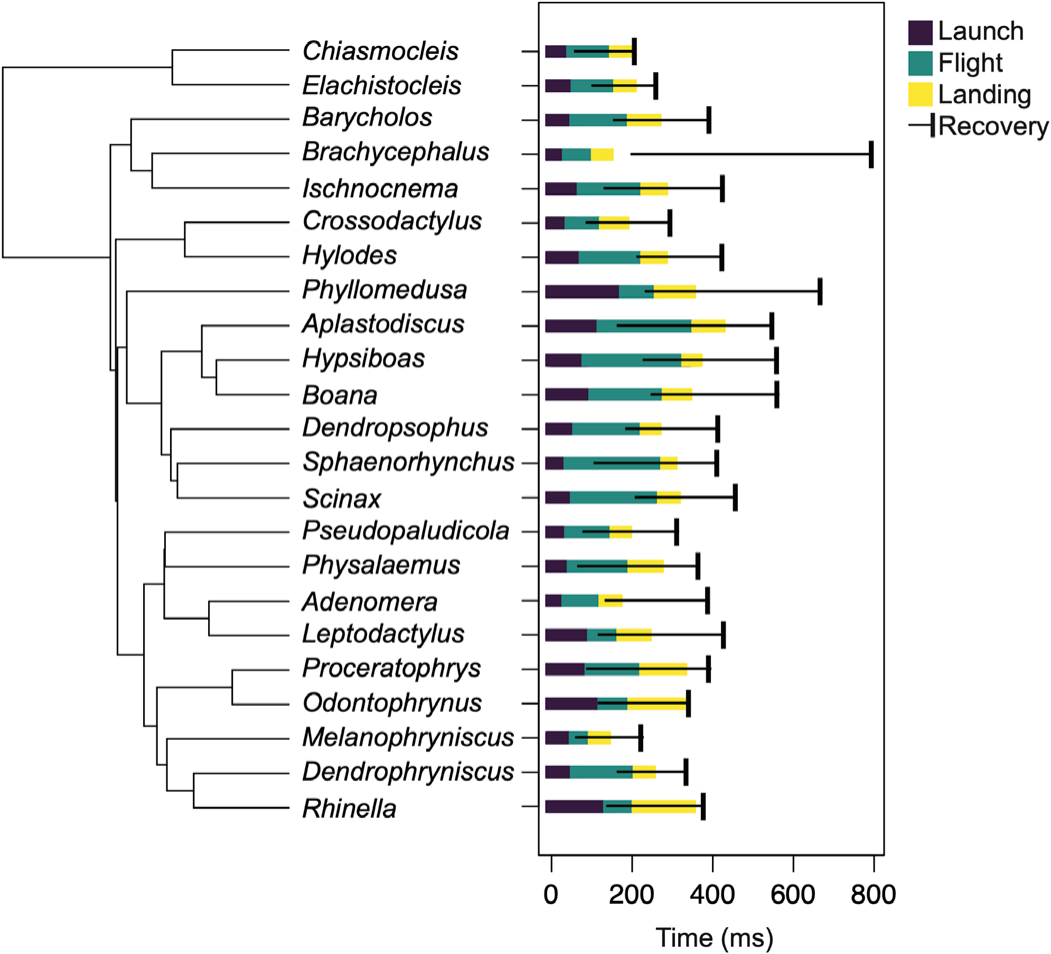
Kinematic timing (in milliseconds) mapped onto a phylogeny of 23 anuran genera (*48*). Phases included launch [beginning of movement (BM) to toe off (TO)], flight [TO to landing contact (LC)], landing [LC to end of forward progress (EFP)], and recovery [beginning of hindlimb flexion (HF) to end of movement (EM)]. Note the delayed hindlimb recovery in *Brachycephalus*, which does not begin until after EFP. The tree frog *Phyllomedusa* also exhibits a relatively long cycle duration. However, it uses derived landing behavior that involves stretching out the limbs to make better contact with adhesive toe pads (*54*). See data S4 for SDs.

Of the 23 genera examined, 22 had mean hindlimb recovery onsets that were negative, indicating that recovery was initiated before the end of landing (Fig. 4 and data S4). The only exception was *Brachycephalus* (*n* = 16; mean = 67.06 ms). To assess the role of phylogeny in hindlimb recovery, we tested for a phylogenetic signal in our dataset. We found no significant phylogenetic signal (*K* = 0.68, *P* = 0.5) and therefore conducted additional analyses without regard to phylogeny.

We used generalized linear mixed models (LMMs) to examine the effects of morphological, ecological, and kinematic variables on recovery delay. Analysis of deviance for hindlimb recovery was significant for the type of landing contact (LC), cycle duration, horizontal range, and body size [snout-vent length (SVL)], as well as the interactions between cycle duration and horizontal distance and habitat type and SVL (Table 3). The best LMM candidate model based on the Corrected Akaike Information Criterion (AICc) (*23*) was the one that included SVL only, indicating that body size is of primary importance in predicting the timing of hindlimb recovery during landing, consistent with our hypothesis of a size-related constraint on vestibular function (Table 4).

**Table 3.** Analysis of deviance results for the best selected model by testing the effect of the type of limb contact, cycle duration, horizontal range, habitat, body size (SVL), and the interactions between cycle duration and horizontal range and habitat and SVL on the probability of mid-flight hindlimb recovery.

	**Chi-square**	**df**	***P***
Contact type	44.97	3	<0.001
Cycle duration	59.20	1	<0.001
Horizontal range	13.86	1	<0.001
Habitat	0.23	1	0.62
SVL	11.73	1	<0.001
Cycle duration:horizontal range	5.82	1	<0.01
Habitat:SVL	27.98	1	<0.001

**Table 4.** Best-ranked linear mixed models based on AICc testing the effect of habitat variables, body size, and their interaction in predicting the occurrence of mid-flight recovery.

**Model**	***K***	**AICc**	**ΔAICc**	**AICc weight**	**Cumulative weight**
~SVL	3	298.05	0.00	0.64	0.64
~Habitat + SVL	4	299.93	1.87	0.25	0.89
~Habitat + SVL + habitat:SVL	5	301.49	3.l43	0.11	1.00
~1	2	323.59	25.53	0.00	1.0

### Alternative hypotheses for pumpkin toadlet jumping behavior

To determine whether there were other plausible explanations for the unusual jumping behavior of *Brachycephalus*, we evaluated a set of additional hypotheses that might be expected to produce similar results. These are broadly grouped into three categories: hindlimb morphology, behavioral compensation, and defensive behavior.

### Hindlimb morphology

The unusual hindlimb morphology of *Brachycephalus* may be a factor in producing rotational motion at the launch. Miniaturization in amphibians is often characterized by digit reduction due to developmental truncation (*24*). In *Brachycephalus*, the number of functional digits of the foot is reduced from five to three. The broadest part of the foot is thought to play an important role in anuran jumping by stabilizing the launch and allowing continuous adjustments of balance (*25*). Reduction in foot width may reduce lateral stability, contributing to a greater propensity to roll and yaw. In addition, reduced plantar surface area might increase the likelihood of slipping, which would result in uncontrolled jumps (*26*). Nevertheless, we only observed slipping in 15 of 138 jumps (~10%) across the four *Brachycephalus* species, which was comparable to our observations of jumping in frog species without digit reduction.

*Brachycephalus* is also characterized by short hindlimbs, which provide little time for balancing adjustments to be made before loss of contact with the ground during the launch. Mean launch duration in *Brachycephalus* was among the lowest in our sample of jumping kinematics in 23 anuran genera (mean = 41.46 ms; data S4). The short propulsive phase may make synchronizing hindlimb extension more difficult which could contribute to rolling or yawing rotation (*27*).

### Behavioral compensation

Maintaining extended hindlimbs throughout the flight phase, rather than protracting them and rotating in mid-flight like most frogs, may also represent a type of behavioral compensation. Hindlimb extension throughout the flight phase is ubiquitous during jumping in *Brachycephalus*. Maintaining an extended posture throughout the jump obviates the need for vestibular feedback and provides the added benefit of slowing intrinsic rotation of the body about the pitch axis by increasing the moment of inertia, in the same way that figure skaters reduce their angular velocity when spinning by extending their arms. Slowing pitching rotation may reduce the likelihood that the frog will land in an inverted posture. Despite this, *Brachycephalus* landed on their backs in more than one-third of jumping trials, although this was often attributable to rotation about the roll axis.

### Defensive behavior

Many leaf litter frogs engage in unusual defensive behaviors to avoid predation. Several species found in the same habitat as *Brachycephalus* exhibit a derived stiff-legged landing posture that has been interpreted as an attempt to resemble a leaf (*28*, *29*). These species have leaf-like camouflage on their backs and remain stationary after landing for as long as 30 min (*28*). Most *Brachycephalus* species are aposematically colored, although a few are cryptic. We included both types in our study but never observed an extended period of immobility after landing in any species that we examined. Rather, individuals initiated hindlimb recovery soon after landing.

Another defensive behavior that could potentially explain the inverted posture of *Brachycephalus* is thanatosis or death feigning. *Ischnocnema*, the sister genus to *Brachycephalus*, is known to engage in this behavior when disturbed to avoid predation. It involves the frog turning onto its back with arms directed upward and hindlimbs partially extended. The individual then remains motionless for an extended period of time (~2 min) (*30*). Thanatosis has also been documented in *Brachycephalus actaeus* (*31*). However, we did not observe it in any of our *Brachycephalus* spp., and inverted landings were soon followed by a righting attempt.

Last, variable escape trajectories are a commonly used tactic used by prey species to confuse predators (*32*). Escape direction was broadly predictable in *Brachycephalus*, generally occurring along a direct line away from the investigator. Variability in posture could be a strategy for confusing predators. However, any mechanical energy used to rotate the body reduces the amount of energy that is directed toward forward movement (*33*). This would be counterproductive because the individual presumably benefits by maximizing the distance between itself and the predator. Delayed hindlimb recovery also makes little sense in predator avoidance, apart from camouflage, because it impedes the ability to make a subsequent escape jump.

### Vestibular dysfunction best explains pumpkin toadlet jumping behavior

Despite the potential for hindlimb morphology, behavioral compensation, and defensive behavior to explain some aspects of jumping behavior in *Brachycephalus*, none explains the full set of observations as well as a loss of vestibular feedback. In light of this, we consider vestibular dysfunction to be the most comprehensive and parsimonious explanation. However, it should be recognized that these hypotheses are not mutually exclusive, and it is possible that some contribute to the observed landing behavior.

In summary, multiple lines of evidence indicate that the uncontrolled landing behavior of pumpkin toadlets results from a size-related constraint on the dimensions of the semicircular canals that has resulted in a loss of vestibular function (i.e., sensory feedback) due to Poiseuille’s law. As a physical constraint, this phenomenon should not be restricted to this clade. Consequently, we expect that uncontrolled jumping behavior will characterize other unrelated miniaturized frog taxa. In addition, a loss of vestibular function should have an impact on other behaviors that involve rapid movement, including feeding and other locomotor modes (*14*, *34*). For example, the vestibular system is also important in quadrupedal locomotion. Experimental ablation of the vertical semicircular canals in frogs was found to have minimal effects during slow crawling but was more pronounced during rapid crawling (*21*). Thus, vertebrates with small labyrinths might compensate behaviorally for reduced sensitivity to angular acceleration by moving slowly and cautiously (*13*, *14*). This is the case for miniaturized *Brachycephalus* (Brachycephalidae) (*35*) [see video S1 in (*36*) to view slow walking behavior in *Brachycephalus ephippium*], *Euparkella* (Strabomantidae) (*37*), *Eleutherodactylus* (Eleutherodactylidae) (*38*), and five recently described miniaturized frogs from three different genera in Madagascar (Microhylidae) (*39*). Thus, we expect that slow walking as the dominant mode of locomotion will be a common feature in many miniaturized frog species, which may partly explain their low vagility and microendemism (*37*).

Uncontrolled jumping with delayed hindlimb recovery and slow walking presumably place miniaturized frogs at greater risk of predation. Thus, we expect that miniaturization in frogs will often be accompanied by antipredator defense strategies such as toxicity, osteoderms, bony plates, aposematic coloration, or camouflage, all of which are seen in *Brachycephalus* (*35*, *40*–*43*). Given the overwhelming importance of scaling in determining fundamental aspects of organismal design, it is likely that future studies of locomotion in miniaturized frogs will reveal additional adaptations and tradeoffs that will further inform our understanding of life at the lower limits of vertebrate body size.

## MATERIALS AND METHODS

### Collection and maintenance of animals

Frogs were collected under permit SISBIO #10277-1, and experiments were carried out in accordance with the Animal Use Ethics Committee of the Universidade Federal do Paraná, Brazil. Frogs were collected during three periods: (i) September 2015 to April 2016, (ii) September 2016 to January 2017, and (iii) September to October 2018. Localities include sites in the Brazilian states of Goiás and Paraná: (i) Reserva do Cachoeira–Antonina, (ii) Morato Salto Natural Reserve–Guaraqueçaba, (iii) Serra State Park of Baitaca–Quatro Barras, (iv) Campina Grande do Sul, (v) Piraquara, and (vi) Vila Velha. Animals were located through both visual and auditory encounters. Animals were placed in plastic bags with natural substrates, which included soil, rocks, or leaves depending on the organism’s preference and transported to the Universidade Federal do Paraná for study. Animals were maintained in polystyrene coolers with natural substrates that included prey. Deionized water was added ad libitum, to ensure animals did not become desiccated. Following the study, animals were returned to the location where they were collected. Our analysis of vestibular shape based on CT scans relied on preserved specimens in existing scientific collections.

### Microtomography

We produced high-resolution microCT scans of 151 specimens (representing 147 anuran species) at the University of Florida’s Nanoscale Research Facility using a Phoenix V|Tome X M nanoCT system. X-ray voltage, current, and detector capture time were optimized for each specimen to maximize voxel resolution and signal. Radiographs were converted into tomograms using Phoenix Datos R, and endocasts of the semicircular canals were segmented using VGStudioMax 3.5 (Volume Graphics, Heidelberg, Germany). We estimated circuit radius (*R*) by fitting a curve to a series of points placed along the midpoint of the entire canal and measuring the distance to the center of the torus (Fig. 1). This measurement was repeated three times and then averaged. Lumen radius (*r*) was estimated as the distance from the outer margin of the endocast to the center of the endocast at its narrowest point (minimum circumference). Because it was not possible to visualize the thin ducts of the inner ear in our CT scans, our measure of *r* is based on the radius of the bony labyrinth. This is an overestimate that does not necessarily reflect the lumen size of the enclosed membranous duct through which the endolymph flows during movement (*8*). Nevertheless, the bony canal represents the maximum boundary for *r*. The actual lumen radii are even narrower, and the semicircular canals are less sensitive than our measurements indicate, which further supports the hypothesis that vestibular function is impeded in the smallest frogs.

### Allometry

In the four cases with more than one record per species, we used the mean for the species in downstream analyses. We estimated the slopes of the relationships between the tested variables using phylogenetic RMA regression, as implemented in the phyl.RMA function in “phytools” 0.4-60 (*44*). In some cases, species in our dataset were not included in the available phylogenies (*45*). We used the following approach to maximizing the phylogenetic coverage of our tested species. If there was only one species in a given genus, then we simply replaced its name with a randomly chosen one from the same genus on the phylogeny. This should provide identical results, as long as the genus in question is monophyletic. If more than one species was not available from a given genus, then we randomly selected the same number of species among those present in the tree. These procedures were repeated for 100 alternative topologies to account for phylogenetic uncertainty.

### Jumping behavior

Frogs from four *Brachycephalus* spp. (*B. brunneus*, *B. coloratus*, *B. pernix*, and *B. sulfuratus*) and *I. henselii* were filmed jumping in the laboratory with a dorsally positioned high-speed video camera (Chronos 1.4) at 1057 fps with a mirror angled at 45°, providing simultaneous dorsal and lateral views. Digitizing was done using the DLTdv7 application in MATLAB R2020a with an eight-point calibration cube (*46*). We digitized two landmarks in both camera views to generate three-dimensional coordinates for the tip of the snout and the tip of the urostyle. Body angle was calculated as the angle formed by a line connecting these two landmarks and the horizontal, which was determined by projecting a point in the *y* direction. Data processing was done with Biomechanics Toolbar v1.02 (Jos Vanrenterghem) in Microsoft Excel 2019. We applied a fourth-order zero-lag Butterworth filter to the three-dimensional coordinates using a sampling frequency of 100 Hz and a low-pass cutoff frequency of 10 Hz based on a residual analysis (*47*). Angular velocities and accelerations about the pitch axis were calculated as the first and second derivatives of angular displacement.

### Hindlimb recovery

The timing of jumping kinematics was examined by filming 23 anuran genera in the laboratory with high-speed video (Canon PowerShot SX50 HS) at 240 fps, next to a mirror angled at 45°. Video sequences were analyzed in Kinovea v.0.8.25 to identify key kinematic events and durations of jump phases. We pruned the phylogeny (*48*) according to our species list and estimated phylogenetic signal with Blomberg’s *K* using the phylosig function in the R package phytools (*44*, *49*). Genera were categorized by habitat type as (i) terrestrial fossorial, (ii) terrestrial nonfossorial, (iii) semiaquatic, (iv) arboreal open canopy, (v) arboreal bushes, and (vi) arboreal high canopy (*50*).

Six kinematic events were identified from each video: beginning of movement (BM), toe off (TO), LC, beginning of hindlimb flexion (HF), end of forward progress (EFP), and end of movement (EM). These events were used to identify the following phases of the jump: (i) launch, measured from BM to TO; (ii) flight, measured from TO to LC; (iii) landing, measured from LC to EFP; and (iv) recovery, measured from HF to EM (*17*). The recovery phase was generally initiated during flight and extended beyond the landing phase, as the frog recovered its limbs to the starting position. The timing of the onset of recovery was measured relative to EFP, which was assigned as time zero. Recoveries initiated before the end of landing were given negative values, whereas those initiated after the end of landing were given positive values.

Recovery delay was treated as a binary variable, with 0 representing jumps with a delayed recovery and 1 representing jumps with recovery beginning during the flight phase. Genus was considered a random factor. Predictors included SVL, cycle duration, horizontal distance, type of LC (e.g., forelimbs first, belly, and hindlimbs first), habitat type, and their interactions. Cycle durations were log-transformed before analysis. Models were fit and analyzed using the glmer function in the R package lme4 (*51*). Model selection was based on the AICc (*23*, *52*) using the R package AICcmodavg (*53*).

## Notes

View/request a protocol for this paper from *Bio-protocol*.
