# Supplementary material for: Semicircular canal size constrains vestibular function in miniaturized frogs
Source: Sci Adv. 2022 Jun 15;8(24):eabn1104. doi: 10.1126/sciadv.abn1104 (PMC9200278; doi:10.1126/sciadv.abn1104)
Supplement: Supplementary file 1 — Fig. S1 [file sciadv.abn1104_sm.pdf]

Science Advances
AAAS

Supplementary Materials for

Semicircular canal size constrains vestibular function in miniaturized frogs

Richard L. Essner Jr. *et al.*

Corresponding author: Richard L. Essner, ressner@siue.edu; Marcio R. Pie, piem@edgehill.ac.uk

Sci. Adv. **8**, eabn1104 (2022)
DOI: 10.1126/sciadv.abn1104

The PDF file includes:

Fig. S1
Legends for movies S1 to S6
Legends for data S1 to S4

Other Supplementary Material for this manuscript includes the following:

Movies S1 to S6
Data S1 to S4

Fig. S1.

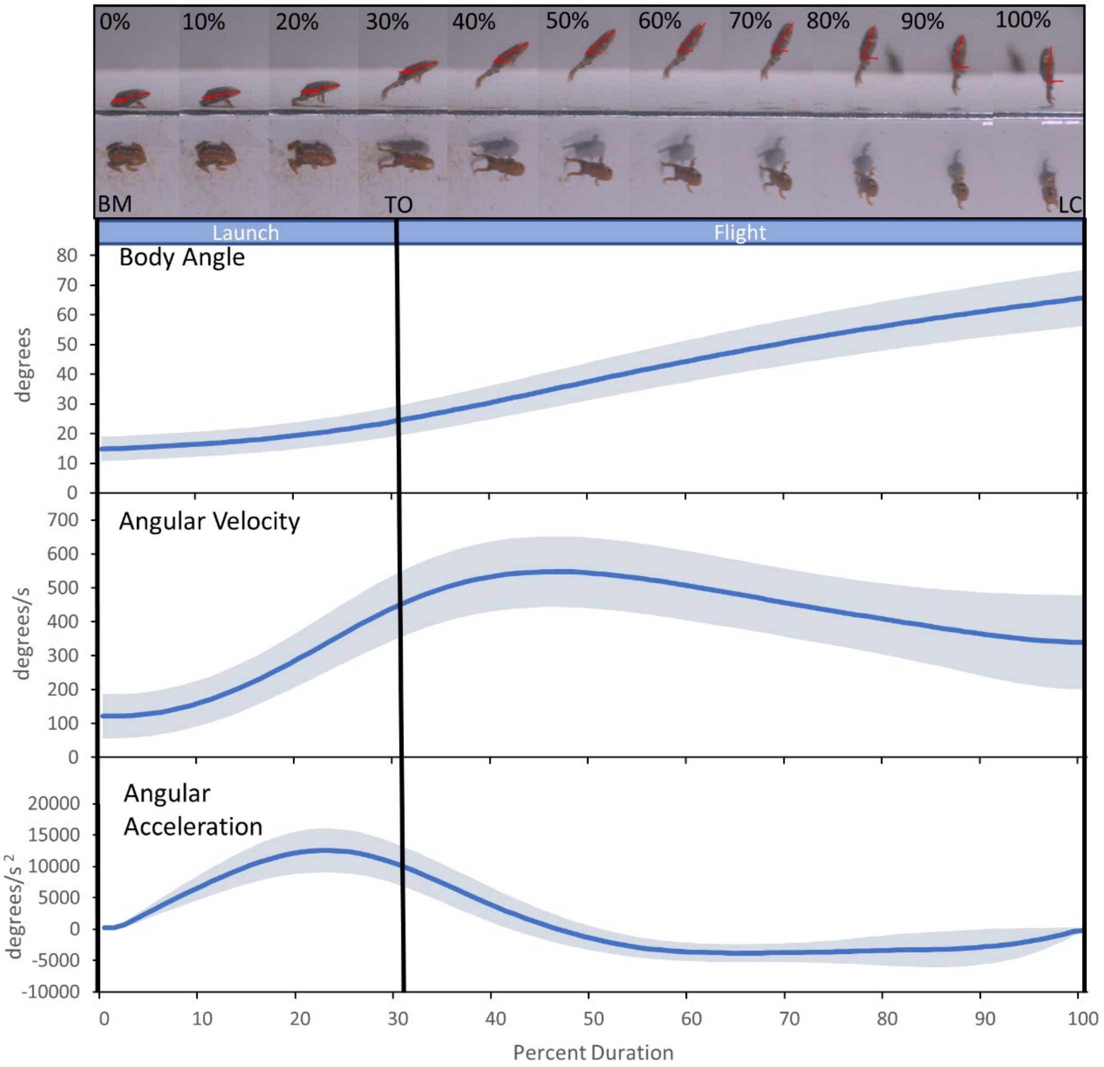

Fig. S1. Mean kinematic profile, angular velocity, and angular acceleration of the body angle during pitching rotation in *Brachycephalus pernix* (*n* = 8). Trials are scaled to 100% duration of the launch + flight phases. Body angle (red lines overlaying photo sequence) was calculated as the angle formed by a line connecting the tip of the snout to the tip of the urostyle and the horizontal. This individual approached the maximum angle of 90° that marked the limit of what we could digitize with our setup. Individuals frequently rotated beyond this angle and landed on their backs (movie S6). Key kinematic events include BM (beginning of movement), TO (toe off), and LC (landing contact). Gray area indicates 95% confidence intervals. During the launch, mean body angle increased from 15 ± 2° at BM to 25 ± 3° at TO (mean ± SEM), and reached a peak of 66 ± 5° at LC. Angular velocity increased gradually during the first part of the launch and then more rapidly, with a peak angular acceleration of 1.3 x 10^4 ± 1,762° s^{-2} occurring at 23% duration, prior to TO. Angular velocity continued to increase briefly after TO, possibly due to hyperextension at the iliosacral joint, reaching a peak of 548 ± 53° $^{s-1}$ at 46% duration. It then decreased gradually until LC. Angular acceleration continued to decrease as angular velocity decreased. It crossed zero and became negative at 47% duration and approached zero again prior to LC, as body rotation neared constant angular velocity.

Movie S1. (separate file)
Ischnocnema henselii exhibiting a typical anuran jump with aerial hindlimb recovery and forelimb protraction.

Movie S2. (separate file)
Brachycephalus brunneus exhibiting uncontrolled aerial rotation with delayed hindlimb recovery.

Movie S3. (separate file)
Brachycephalus coloratus exhibiting uncontrolled aerial rotation with delayed hindlimb recovery.

Movie S4. (separate file)
Brachycephalus pernix exhibiting uncontrolled aerial rotation with delayed hindlimb recovery.

Movie S5. (separate file)
Brachycephalus sulfuratus exhibiting uncontrolled aerial rotation with delayed hindlimb recovery.

Movie S6. (separate file)
Brachycephalus pernix exhibiting excessive pitching resulting in a backwards somersault.

Data S1. (separate file)
Body size and semicircular canal measurements based on 3D endocasts of the inner ears of 147 anuran species from 54 families.

Data S2. (separate file)
Angular kinematic data for *Brachycephalus pernix*.

Data S3. (separate file)
List of anuran species included in kinematic timing analysis.

Data S4. (separate file)
Kinematic timing data for 38 anuran species representing 23 genera and nine families (mean ± standard deviation).
